# Translating the Cognitive Model of PTSD to the Treatment of Very Young Children: A Single Case Study of an 8‐Year‐Old Motor Vehicle Accident Survivor

**DOI:** 10.1002/jclp.22449

**Published:** 2017-04-17

**Authors:** Benjamin Goodall, Isobel Chadwick, Anna McKinnon, Aliza Werner‐Seidler, Richard Meiser‐Stedman, Patrick Smith, Tim Dalgleish

**Affiliations:** ^1^ Medical Research Council Cognition and Brain Sciences Unit; ^2^ Macquire University; ^3^ Black Dog Institute; ^4^ University of East Anglia; ^5^ Kings College London; ^6^ Cambridgeshire and Peterborough NHS Foundation Trust

**Keywords:** Post‐traumatic stress disorder, children, cognitive model, trauma‐focused cognitive behavioral therapy

## Abstract

Posttraumatic stress disorder (PTSD) is a clinical condition that occurs after a discrete traumatic event, such as an accident or assault. Research into PTSD has primarily been adult‐focused; however, there is a growing body of evidence evaluating the theory and treatment of PTSD in young children. Consequently, cognitive behavior therapy (CBT) interventions for PTSD in youth have been developed that focus on 3 core components of the cognitive model–a disorganized *memory* of the trauma, maladaptive appraisals of the trauma and its effects (*meanings*), and dysfunctional coping mechanisms (*management*). Here, we describe the extension of this treatment approach (termed CBT‐3M) to very young children (3–8 years) through the case of Dylan, an 8‐year‐old motor vehicle accident survivor. This serves as an illustration of the underlying theory and its successful application. Further work is intended to provide evidence of the efficacy of this treatment via an ongoing treatment trial.

Posttraumatic stress disorder (PTSD) is a condition that occurs in the aftermath of a discrete traumatic event, for example, an accident, assault, or disaster. PTSD is characterized by disabling symptoms from four clusters; reexperiencing (e.g., flashbacks and nightmares about the trauma; avoidance (e.g., efforts to not think about or talk about the trauma); negative cognitions and mood (e.g., a persistent and distorted sense of blame of self or others to an inability to remember key aspects of the event); and hyperarousal (e.g., anger outbursts and hypervigilance for threat; *Diagnostic and Statistical Manual of Mental Disorders 5th edition [DSM‐5];* American Psychiatric Association [APA], 2013). Traumatic events with the potential to lead to PTSD are experienced by up to two thirds of children by 16 years of age (Copeland, Keeler, Angold, & Costello, 2007), including in preschool and early school years, even when excluding abuse. However, the DSM‐5 was the first edition to include tailored criteria capturing the differential presentation of the disorder in very young children younger than 6 years old.

The inclusion of these preschool criteria within the *DSM‐5* follows work by Scheeringa and colleagues (e.g., Scheeringa, Peebles, Cook, & Zeanah, 2001; Scheeringa, Zeanah, & Cohen, 2011), who developed an alternative PTSD algorithm for young children. PTSD criteria prior to the publication of the *DSM‐5* had been adult‐oriented, leading to an underdiagnosis of PTSD in youth (Scheeringa, Zeanah, et al., 2011). Scheeringa's alternative algorithm required only one symptom from each of the then three symptom clusters in the *DSM‐IV* (APA, 1994), thereby critically reducing the avoidance criterion (the biggest barrier to diagnosis in youth) from three symptoms to one.

In addition, the alternative algorithm took account of how children's limited cognitive and expressive language skills (Scheeringa, Zeanah, Drell, & Larrieu, 1995) constrained their ability to describe their subjective experiences, with a consequent focus on behavioral markers of distress. Young children also typically display a different range of behaviors than adults, including increased nightmares not related to the trauma; extreme difficulty falling asleep or frequent night waking, requiring significant increase in parental involvement; refusal to eat or trouble keeping food down; and changes in responsiveness to an adult's attempts to calm them that may include responding with heightened irritability, fearful expressions, crying, or blank expressions under circumstances that do not normally produce these effects (that is, face‐to‐face play or efforts to comfort; Carpenter & Stacks, 2009).

According to these new criteria, a significant proportion (10% to 40%) of younger children who are exposed to nonabuse traumas are severely affected and go on to develop PTSD (Levendosky, Huth‐Bocks, Semel, & Shapiro, 2002; Meiser‐Stedman, Smith, Glucksman, Yule, & Dalgleish, 2008). For example, 10% to 14% of 3‐ to 8‐year‐old children were diagnosed with PTSD when assessed with the developmentally appropriate criteria proposed by Scheeringa 6 months after presenting at a U.K. emergency department after acute trauma (Meiser‐Stedman et al., 2008). There is evidence that if left untreated, PTSD in children and young people can lead to a chronic course lasting a number of years (Yule et al., 2000). In support of this, 10% to 15% of 3‐ to 8‐year‐olds have previously been found to have PTSD 3 years after a trauma, which is in line with the consensus in the field that chronic PTSD in young children after discrete traumas shows little spontaneous remission (Meiser‐Stedman et al., 2008). Part of the difficulty is that experiencing trauma at a young age can disrupt typical developmental processes: There may be increased difficulty coping with frustration, bouts of intense fear, sleep disturbances, regression in developmental achievements, social withdrawal, and generally higher levels of mood and behavioral problems in comparison to control groups (Lieberman & Knorr, 2007), any or all of which can lead to cumulative difficulties if left untreated.

Archetypal PTSD symptoms including intrusions, avoidance, negative mood and cognition, and hyperarousal after a trauma are considered to be a normal response to an abnormal event; however, it is the maintenance of such symptoms that characterizes PTSD. There are a number of theories that attempt to explain how such symptoms coalesce over time to form PTSD. Here, we focus on the cognitive model (see Dalgleish, 2004) because it forms the theoretical backbone of our treatment. What follows is a summary of the cognitive model and how it explains the development and maintenance of PTSD.

## Cognitive Theories of PTSD in Adults and Youth

PTSD as an anxiety or stress disorder presents an interesting theoretical challenge. Such disorders are generally seen as the result of misappraisals leading to the interpretation of an imminent or future threat. However, in PTSD the anxiety concerns a memory of what has already occurred. How can we resolve this discrepancy? Ehlers and Clark (2000) suggest that for PTSD to occur, the individual needs to interpret an element of the traumatic experience as being ongoing––in that way, it represents a current and future threat. For this to happen, they suggest that two key processes need to be present: *A distorted representation of the trauma memory and its link to other autobiographical memories; and a pattern of maladaptive appraisals of the trauma or its sequelae*. The sense of current threat is then accompanied by the key symptom clusters of reexperiencing, avoidance, negative cognitions and moods, and hyperarousal.

A central tenet of cognitive theories of PTSD is that the nature of the trauma memory is critical to the development of the disorder (Dalgleish, 2004). Those suffering with PTSD often have difficulty retrieving information about the traumatic event, and the recollection is often disjointed and fragmented (Brewin, Dalgleish, & Joseph, 1996). Second, as we have seen, survivors often experience high levels of intrusive memories that are emotionally laden. Cognitive theorists (e.g., Brewin et al., 1996) suggest that this apparent paradox is a function of two different retrieval pathways of information from the autobiographical memory base. Intentional retrieval is thought to depend on organized verbal (propositional) representations of the trauma. Intentional retrieval can therefore be disrupted when the trauma memory is not sufficiently elaborated and poorly contextualized in time and place. Involuntary intrusive recollections, in contrast, reflect the operation of sensory‐based trauma representations with minimal propositional content that can be activated by encountering sensory cues reminiscent of the trauma—triggers–in the environment (Brewin et al., 1996; Dalgleish, 2004).

The second key process Ehlers and Clarke (2000) identify is the presence of maladaptive appraisals; they suggest that an individual with PTSD is unable to appraise the event and its implications as time‐limited, thus supporting a sense of current threat. These perceived ongoing threats can be external or internal––for example, the world is a more dangerous place, or altered beliefs about one's ability to manage in stressful scenarios. With regard to the traumatic event itself, common misappraisals may also be external or internal. The individual might make an overestimation of the prevalence of that event and therefore the perception of future risk. Consequently, he or she might develop a strategy for managing that risk that actually maintains the fear via avoidance, for example, avoiding the activity during which the event occurred due to unrealistic beliefs about the risk of the event recurring. Alternatively, the individual might believe that because he or she experienced the trauma, and not somebody else, that “bad things happen to me.”

In terms of appraisals of feelings surrounding the event, it is common for people to develop thoughts that certain of these emotions (e.g., guilt, shame) confirm that they were in fact responsible for the event (Lee, Scragg, & Turner, 2001). Alternatively, survivors may develop thoughts about their actions that change their view of their ability to resolve or manage stress; in other words, not coping with this experience means a fundamental problem with coping exists.

Those with PTSD often see their symptoms as a sign that they have changed or that their mental health has been permanently affected, rather than viewing these responses as common and understandable reactions to a stressful event. These appraisals can very often lead to maladaptive coping strategies.

Those with PTSD will also often interpret others’ actions or signs of support as confirmation of some internal detriment. For example, those not wishing to discuss the event for fear of upsetting the victim may have their actions interpreted as a sign of not caring or that the event was partially the victim's fault (Ehlers & Clark, 2000). Again, such interpretations can lead to dysfunctional strategies such as social withdrawal or avoidance of conversations, which in turn can reduce the opportunity for therapeutic reliving or positive feedback to counter these negative appraisals.

Negative appraisal is associated with certain emotional responses, and Ehlers and Clarke (2000) go into detail about how perceived danger may lead to augmented fear, violation of personal rules may lead to anger, and appraisals about responsibility to shame or guilt. It is acknowledged that a number of negative emotions will occur over time because the appraisals will be activated in different situations and times and because personal conviction in their veracity will fluctuate.

There is now a substantive body of research supporting the cognitive model of PTSD in adults (see Brewin & Holmes, 2003, and Dalgleish, 2004, for reviews). Central to the current article, Meiser‐Stedman has advanced the model for youth (Meiser‐Stedman, 2002) using the core elements that Brewin et al. (1996) and Ehlers and Clark (2000) highlight. Subsequently, there has been a growing research interest examining the cognitive model of PTSD in younger populations.

For example, in a study of 93 children aged between 10 and 16 years presenting at an emergency department after either an assault or a motor vehicle accident, Meiser‐Stedman, Dalgleish, Smith, Yule, and Glucksman, (2007) found that a diagnosis of acute stress disorder (ASD) was associated with a greater sense of threat at the time of the trauma, the presence of relatively more sensory‐laden memories of the trauma, and higher scores on a range of trait cognitive style measures indexing the presence of dysfunctional posttrauma appraisals. The development of ASD was mediated by the presence of more sensory‐laden memories and there was a trend for dysfunctional appraisals to mediate this relationship as well (Meiser‐Stedman et al., 2007). This is important because ASD has the same core symptom clusters of PTSD but is diagnosed within the first four weeks posttrauma. A follow‐up study went on to show that distorted memory representations and dysfunctional appraisals mediated the development of later PTSD in the 10‐ to 16‐year‐olds (Meiser‐Stedman, Dalgleish, Glucksman, Yule, & Smith, 2009).

Based on these theoretical and empirical evaluations of the cognitive processes implicated for PTSD in youth, a cognitive therapy intervention tailored for young people –trauma‐focused cognitive therapy (TF‐CT)–was developed (Smith, Perrin, Yule, & Clark, 2010). TF‐CT focuses on the three “Ms” the cognitive model highlights as central to PTSD in youth: distorted memory representations *(memories)*, maladaptive cognitive appraisals *(meanings)*, and impoverished coping *(management)*. The first trial to evaluate this age‐appropriate version of TF‐CT recruited 24 youth between 8 and 18 years of age who met *DSM‐IV* criteria for PTSD after single incident trauma, and they found that 92% of those who received a 10‐week course of TF‐CBT no longer met diagnostic criteria, versus 42% of a waitlist group (Smith et al., 2007). In addition to this, it was noted that trauma‐related misappraisals mediated the effect of TF‐CBT relative to the waitlist control on PTSD in children and adolescents (Smith et al., 2007). A second trial (Meiser‐Stedman et al., in press) replicated these findings in youth across a similar age range but this time in the acute posttrauma phase.

These two trials in older children of the cognitive approach to intervention (TF‐CT) are encouraging and the results are in line with a broader literature endorsing cognitive‐behavioral approaches (Cary & McMillen, 2012). However, to date, there has been only one small pilot clinical trial examining any form of CBT in younger children with symptoms, but not a diagnosis, of PTSD, in which children were allocated either to receive 12 sessions of manualized CBT or to a waitlist control group (Scheeringa, Weems, Cohen, Amaya‐Jackson, & Guthrie, 2011). The results of this study, conducted in New Orleans, supported the feasibility and effect of a developmentally tailored form of CBT, with a large effect size and treatment gains maintained at the 6‐month follow‐up. However, it is unclear whether the results from this study are generalizable, particularly within the United Kingdom, given that the trial was conducted in an underprivileged, low social‐economic group in an urban American setting (Scheeringa, Weems, et al., 2011 ).

Based on the previous work with children and adolescents, aged 8–17 years, showing that TF‐CT improves symptoms of PTSD, anxiety, and depression, as compared to a waitlist control group, we adapted this program (Smith et al., 2007; Meiser‐Stedman et al., 2016) for use with much younger children (Dalgleish et al., 2015). Adaptations include the use of cartoons, pictures, and toys to illustrate the process and develop the narrative. Worksheets also include simplified concepts, simple language, and child‐friendly depictions very young children can understand. This trial (Dalgleish et al., 2015) is designed to assess the efficacy of this intervention–termed *CBT‐3M* to reflect the clinical focus on memories, meanings, and management–for children aged 3–8 years with PTSD after single incident traumas and is being compared to a treatment as usual (TAU) group. This trial will also assess a child's willingness to engage in therapy and undergo this emotionally demanding work.

To illustrate the components of this novel treatment approach, we present a case study of an 8‐year‐old male survivor of a motor vehicle accident who had a diagnosis of PTSD according to *DSM‐5* preschool criteria.

## Case Illustration

### Presenting Problem and Client Description

Dylan was 8 years of age at the time of treatment. He had been involved in a car crash that occurred not far from his family home. He was a front‐seat passenger in a vehicle driven by his father, and they were returning home from a soccer practice. At a roundabout, a turning circle directing traffic through, in this instance, a five‐way intersection, Dylan's father slowed down for a vehicle already on the roundabout that had right of way, but the vehicle behind them was travelling too quickly and did not manage to brake in time and there was a collision. Neither Dylan nor his father suffered major injuries. Dylan's father, however, did suffer a minor injury to his knee after it hit the steering column in the impact. The car Dylan was traveling in was not structurally damaged, the air bag did not deploy, and the vehicle was driveable immediately after the event. After the incident, Dylan's father went to exchange personal and administrative details with the other driver for the purposes of insurance. The other driver was very angry with Dylan's father and a verbal altercation occurred, with the threat of violence. Dylan's father climbed back into the car to drive away and they were promptly pursued by the other driver for a period of time before the other driver pulled off the road, leaving Dylan and his father to proceed with their journey. The incident was reported to the police when they arrived home.

Dylan and his two older siblings lived with their father, who was sole carer for the family after his wife left the family home a number of years previously. Dylan was a talented young athlete, who played soccer for a local team. Dylan's father had a physical health condition; consequently, he did not work but was in receipt of a disability living allowance from the state. His father had no previous history of mental health conditions, though he acknowledged periods of low mood as a consequence of not working and struggling as a single parent to raise three children.

Dylan was referred a year after the collision through the local Child and Adolescent Mental Health Service (CAMHS)–the state‐funded mental health care service in the United Kingdom. He presented with intrusive memories of both the crash and the altercation that followed. Dylan had a very disjointed verbal narrative of the incident and only really understood the end result of the crash (i.e., the damage to the car and the pursuit home). He was reported to be highly anxious at reminders of the incident, from talking about it, to the type of vehicle involved, to the stretch of road, to stories on television with crashes, injuries, or death as part of their content.

Dylan was presenting with great distress at bedtime and was no longer able to go to sleep on his own, insisting on sleeping in his father's bed and taking up to a couple of hours to go to sleep. Nights were frequently disturbed by nightmares, though it was unclear what the content of these were because they were not normally discussed either at the time or the following morning. Dylan was showing more physical aggression than prior to the accident, and this manifested itself both at home and at school. At home, he was frequently getting into fights with his older siblings and lashing out at the slightest provocation. At school, it had been noted that he was no longer concentrating on his school work and was failing to complete work, even with assistance. His frustration with the situation would often lead to outbursts in class, including becoming so worked up that he began overturning tables and trashing a classroom. No one was hurt in the incident; he was left in the classroom as his classmates were removed to safety, rather than trying to isolate him in a distressed state. The school was willing to work with agencies around Dylan to resolve the situation and address his greater needs at the same time.

With regard to comorbidities as well as PTSD, Dylan met the criteria for attention deficit hyperactivity disorder (ADHD), oppositional defiant disorder (ODD), conduct disorder (CD), major depressive disorder (MDD), separation anxiety disorder (SAD), and specific phobia for spiders. These were all assessed using the Diagnostic Infant and Preschool Assessment (DIPA; Scheeringa & Haslett, 2010). There had been a long‐standing concern over ADHD from before the event, with information being exchanged between the school and Dylan's father, and Dylan had subsequently been referred for assessment in CAMHS specifically for this.

### Case Formulation

Having met criteria for PTSD on the DIPA, it was clear that Dylan was suffering with intrusions, avoidance, and hyperarousal as a consequence of the crash. He appeared to have a disorganized understanding of the event and limited capacity to discuss the crash, especially the emotional content of the subsequent intrusions. Except for the ADHD and specific phobia, the comorbidities appeared to have their onset in the weeks after the crash. We hypothesized that these were due to Dylan's difficulties with processing his anger and fear and choosing to lash out as a means to remove himself from feared situations or to prompt contact with his father. For instance, at school, lashing out often led to him to be picked up early by his father, thereby reinforcing the behavior and leading to an entrenched avoidance tactic.

**Figure 1:**
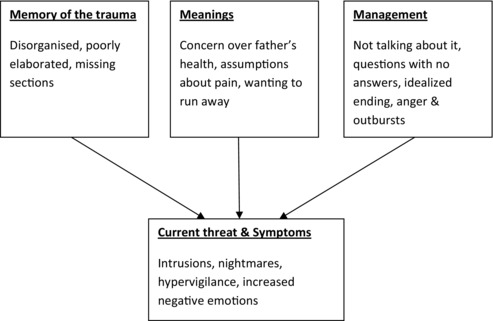
Formulation of Dylan's presentation using the three Ms from the cognitive model of PTSD: Memories, meanings, and management.

As is normal, the formulation guided the goals of treatment, and we aimed to reduce the current threat and symptoms by addressing the three Ms–memories, meanings, and management–of the trauma in line with the cognitive model of PTSD in youth (see Figure 1). Specifically, goals of treatment comprised a reduction in the number and frequency of intrusions and nightmares, a shift in the nature of the appraisals of the trauma (meanings), the adoption of better emotional management strategies (management), and processing of the trauma (memories). We agreed with Dylan and his father that the central focus of the therapy would be to develop a memory narrative by integrating the emotional content of the intrusions and nightmares with the current fragmented story, while discovering and filling in the blanks (memories), before addressing any misappraisals about the trauma itself, Dylan's response to it, or to the consequences arising from the crash (meanings), and integrating these new insights into the evolving narrative. In parallel, we would work with Dylan and his father behaviorally to address any remaining management and coping problems (management).

#### Course of treatment

The CBT‐3M package is a manualized, 12‐session protocol that develops the child's skills to discuss the trauma and accept the need and validity of those discussions. As noted, the manual is based heavily on the treatment developed by Smith et al. (2007) for children aged 8–18 years, but it also incorporates aspects of the treatment devised by Scheeringa et al. (2007) for children with PTSD aged 3–6 years, for example, the initial work on understanding, monitoring, and managing emotions. The manual has been adapted to be developmentally appropriate and consequently there are two versions: one for those children aged 3–4 years, and one for the older age group aged 5–8 years. Both manuals include the same treatment stages but are adjusted to be sensitive to developmental ability.

Treatment begins by focusing on engaging the family, encouraging a return to the family's pretrauma routine and activity level, providing psychoeducation, and normalizing the child's response. Following from this, the child begins work on understanding emotions, discriminating varying levels of emotional intensity, and learning relaxation and imagery‐based anxiety reduction skills. The majority of the treatment is spent on facilitating sufficient procession and elaboration of the trauma memory through the development and evolution of a detailed trauma memory narrative, with a focus on integrating new information so that the memory can be updated. This narrative can be verbal or drawn/painted, or both. The narrative is then organized and elaborated using detailed questioning and with the help of pictures, drawings, or other sources, such as toys. Cognitive therapy techniques are used to examine and change maladaptive appraisals of the event and symptoms (meanings), although in younger children this mostly comprises behavioral techniques. Throughout the memory work, the narrative is continually updated with any new information as it becomes available, which assists in modifying maladaptive appraisals (meanings). A visit to the trauma site or exposure to distressing reminders can be incorporated if indicated as useful and serve to further modify maladaptive appraisals.

Dylan's treatment started with an explanation of the treatment rationale, and an introduction to the core components of CBT‐3M. His father joined for a number of early sessions, with the aim that, as time went on, Dylan and his father would begin to have separate time with the therapist to address problems, discuss next steps, and deal with their individual responses to the trauma. Because the efficacy of CBT‐3M is being assessed, there is limited focus on working with the parent for their mental health needs in relation to the trauma; however, there is an acknowledgement that the parent(s) needs to be prepared for their child's responses to therapy and they often need help or guidance as to how to deal with their own symptoms. If there is clinical need for the parent to have independent therapy as a result of the trauma, then they are advised, encouraged, and supported to access appropriate services.

The initial sessions included a lot of rapport building and normalizing of the response to the trauma. A cartoon, depicting a motor vehicle accident, emotional reaction, therapy, and symptom improvement, was used to normalize the fact that accidents happen, that children get scared, and that they are seen therapeutically with the aim of promoting symptom reduction. Dylan and his father were informed that trauma can happen to anyone, child, adolescent, or adult, and we would all experience some of the symptoms that Dylan was dealing with. These initial sessions also began the process of discussing the trauma, and it is important in this context that the child knows she or he has permission from the adult to discuss the trauma, while at the same time normalizing the discussion of events, emotions, and responses to them. This is also a useful process in helping the parent to see that the trauma can be discussed safely, calmly, and without burdening the child.

**Figure 2:**
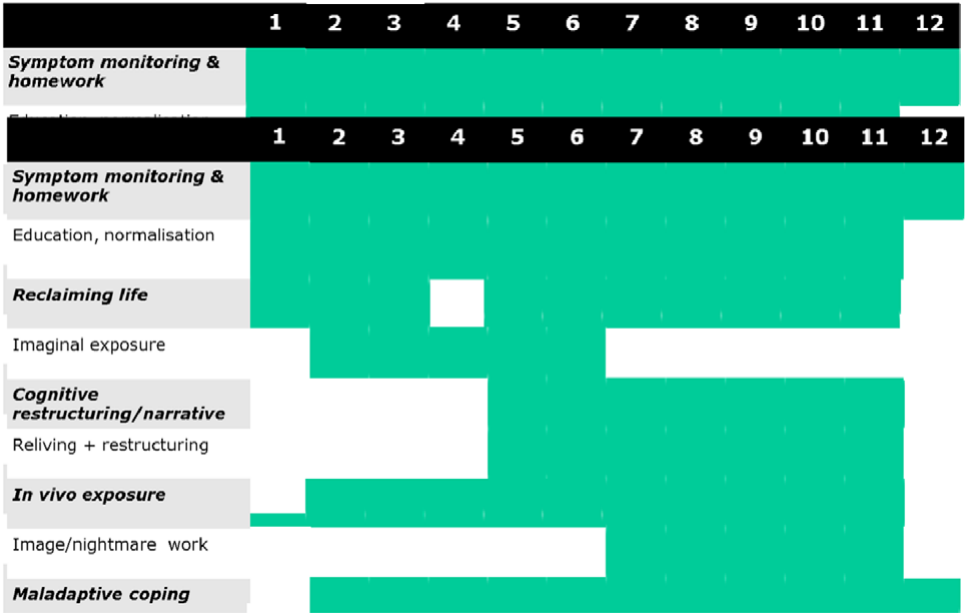
Gantt chart of treatment elements over the course of sessions. *Note*. The Gantt chart is a guide through the stages of treatment; number of sessions for each element is based on the child's ability to complete the relevant section. Also, elements are illustrative, for example, image/nightmare work is required only if clinically indicated.

Dylan came from a home situation that was limited in its discussion of emotions. There was therefore a lot of work initially about recognizing emotions, expressing emotions, and managing extreme emotions. To this end, we introduced methods of relaxation and management, especially progressive muscle relaxation and the use of mental imagery. This is a very good tool for overall management of emotion outside of the therapeutic context, but it is also useful in addressing emotion as it occurs in later therapy sessions when working on the trauma narrative. The relaxation techniques give the child the experience of paying attention to his or her internal emotional state and of observing how relaxation techniques cause a change in these emotions. With the support of the parent, the child is encouraged to use it at home; the parent is also encouraged to use it with the child, and the child is encouraged to teach others in the family the technique, thereby instilling a sense of mastery. This is the first homework within the package of learning and practicing relaxation techniques, and as can be seen from the Gantt chart (Figure 2), practice continues throughout the treatment process.

Given Dylan's separation anxiety and the change in his patterns of play to minimize separation from his father, even within the house, it was deemed important to start in the initial sessions with reclaiming his life via behavioral activation, in parallel with the primary focus on memory work. Having socially isolated himself, Dylan was inadvertently reinforcing a message that the world was different to him before the accident. He was also no longer receiving important feedback that he could manage situations by himself without the need for protection from his father. Introducing new activities (behavioral activation) also sent the message that life continues and that the crash was in the past, beginning the process of creating updated memories postincident.

We therefore scheduled a series of tasks that Dylan and his father could do together, many of which they were already doing but in response to an anxiety or stimulus. By planning these activities in advance, Dylan knew he had dedicated time with his father without the need to compete with his siblings or chores his father needed to do. The first task was simply playing soccer together in the garden, in the street, and then in the park. The idea was to gradually expand Dylan's geographical horizons while also opening him up to spontaneous contact with his friends. This activity was also important in providing feedback that the pain he experienced in the accident was not permanent. He would happily head the soccer ball and experience no pain or side effects in his neck or shoulders as a consequence of doing so.

A core part of the CBT‐3M approach is to use all therapeutic activities to identify dysfunctional meanings surrounding the trauma and its consequences. The behavioral activation work revealed that part of Dylan's concern about separating from his father was a belief that something would happen to his father. We addressed this belief by looking together at tasks that Dylan's father did and how well he managed them. His father had been in a number of altercations over many years prior to the crash. However, he had always been able to walk away from the incidents and we used this fact as an example of his fathers’ skill in looking after himself.

As mentioned earlier, at the start of therapy, there was limited communication involving pertinent family news, health, or emotions between father and son. With so little spoken about, there was a lot of uncertainty on both sides, Dylan's father did not know of his son's concern for him nor the content of Dylan's intrusions and nightmares. Meanwhile, Dylan did not know that his father's health problems were in the past. This often led to a pattern of mutual avoidance, with assumptions that everything was alright or with Dylan filling in blanks. As is often the case, the blanks were often completed with information that was worse than the truth. Consequently, work in the session was carried out with the father to manage conversations and open up previously closed areas. Given the long‐standing nature of this pattern (i.e., predating the trauma), it was hard for Dylan's father to do this, and these changes needed encouragement and support over a number of sessions. However, Dylan's father noticed that, having clarified a few points with Dylan, his older children had also begun to ask questions around the same topics. This promoted further discussion and led to a more open pattern of communication overall where appropriate.

Emerging from this work was the realization that a major worry of Dylan's was his father's ongoing medical condition and the pain his father experienced in the crash. Dylan believed that his father was still carrying injuries from the trauma and that the consequences of the accident were greater than he had been lead to believe. We therefore generated a series of questions that Dylan could ask his father to update his understanding of his father's condition and of the effect of the accident. Part of Dylan's confusion was that–now that he was spending more time with his father–he was alert to his father taking medication to maintain his health. He had thought the medication was linked to the trauma and that the crash had caused his father's illness to recur. We were able to introduce the information that his father was actually doing very well medically but that he required the medication to maintain his health. Dylan asked if it was like taking hay fever medication, which illustrated his integration of this new knowledge into his previous knowledgebase and the search for a meaning that was relevant and meaningful to him. This is an example of using all the information gathered therapeutically to update and restructure the memory and any dysfunctional appraisals.

### Developing the Trauma Narrative

Working on the trauma narrative and associated appraisals took up the majority of the treatment sessions, approximately eight in total. Over the course of the treatment sessions the rationale for the trauma narrative was revisited and discussed in relation to therapeutic gains and changes in understanding of the trauma and its consequences. With a greater level of communication Dylan seemed more comfortable with both the process and content of the narrative; at the start, he was reluctant to elaborate on his thoughts or difficulties, but over the course of treatment, he became more confident and capable in expressing his thoughts especially in integrating new information. He was given options for writing it, drawing it, or talking it through with the therapist recording it. He chose the latter, having initially started to write it down but becoming frustrated with how slow that process was due to his own handwriting. The narrative was recorded verbatim, and printed up at the end of each session so that the new details were integrated ahead of the following session. During the recounting of the narrative, Dylan was encouraged to relay his emotional state using a simple Likert scale of distress: 0 being no distress and 10 being the most he had experienced.

It was clear from the first narrative, detailed below, that there were deficits around the extent of his knowledge of the event and his understanding of the outcomes; clearly, too, there were areas where Dylan's memory was blank.

Well, erm, it was painful. Then I was frightened. It was really a little bit of a hard crash. Well, erm, it, erm, it was really, erm, it was like I was going to have to go to hospital, it literally hurted everywhere. It hit us bad and it looked as if it was to hit the curb bad too. They got out of the car and it, erm, it, it was like they were circling round us and looked like they were going to fight, they were using strong words. It was the first crash I had been in, that is all I can remember.

Together with Dylan we described this first narrative as an incomplete jigsaw puzzle in which he would need to be a detective to hunt down the missing pieces and identify someone who would be able to able fill in the blanks. It is useful to illustrate with an example. In his initial recounting of the narrative–Dylan acknowledged the physical pain as being the worst pain he had ever experienced, but as is common in rear‐end accidents, he was unaware that the impact was about to happen and the likely impact speed and he did not understand why his father had been braking and was unaware of the other driver–it transpired that Dylan had interpreted his injuries, and those of his father, as being serious enough to warrant immediate hospital treatment. However, he had not received any such treatment and therefore felt that there were still unresolved physical problems resulting from this omission.

As the blanks in the account were filled in, Dylan was told that his father had slowed for a vehicle on the roundabout and the vehicle immediately behind that one was an ambulance. Immediately, on hearing this, Dylan was able to clarify that the ambulance was seen twice, which his father confirmed. The ambulance crew had witnessed the accident and had proceeded to go back round the roundabout to see if assistance was required. The ambulance therefore drove past twice, without stopping, because once the crew was aware that everyone was moving unaided, they proceeded on with their original duties. The presence of an ambulance in the vicinity at the time created an assumption in Dylan's mind that he would need to go to hospital and therefore that the injuries were very serious. We were therefore able to integrate this new information and restructure Dylan's narrative, inserting information reflecting his new understanding that the accident was not serious; otherwise, the paramedics would have stopped, rather than departed, once they had checked all were well before returning to their original nonemergency duties.

The following excerpt from the narrative shows its development and elaboration, including new statements that were integrated after the process exemplified above. One of the techniques used to integrate new information was “what I know now” statements, which help to differentiate memory recall from updated information and reappraisals.

Dad and I were coming back from football training. I do not know where the other driver was going, but we were heading home. I was listening to music on the radio and messing about with Dad's phone. I let go of the door handle, and then the van just hit us. It was painful. Everything was painful, especially my neck. I now know that the pain did not last long, and after a couple of days, it had gone completely. Dad was hurt; I did not know where he was hurting. I now know that he only hurt his knee as it banged against the dashboard; I now know that this is fairly common. Then I was frightened. It was really a little bit of a hard crash. It looked as if the airbag was going to go off; the airbag did not inflate. Well, erm, it, erm, it was really, erm, it was like I was going to have to go to hospital; it literally hurted everywhere. I now know that I thought I would need to visit the hospital due to an ambulance on the roundabout; however, once it had seen everyone was moving and okay, it proceeded on its journey. I now know that this is confirmation that no one was seriously injured, as they would not have driven away if someone needed help. I now know that my injuries, and my Dad's injuries, were not severe enough to need hospital attention. I went to the GPs a few days later and everything was fine and I needed no physical treatment.

Dylan acknowledged in a later part of his narrative that he had wanted to run away. He felt he was not too far from his friend's house and he could have made it there to get help. This was later appraised as an indication that he was someone who is unable to cope in stressful situations. It also led to feelings of guilt that he could have gotten help but instead wanted to run away. Identifying new facts and subsequent appraisals helped explain some of Dylan's concerns for his father's safety. We normalized his thoughts at the time about fleeing as being understandable in the face of a new experience that was scary and without a known ending. We addressed and worked on accepting the reality of him as a younger child having no knowledge of such events. We integrated this new information into Dylan's narrative along with information about his father first checking on him and ensuring he was well before exiting the car, the situation coming to an end, him having reached a place of safety, and all the while his father being able to protect him and get him there.

Dylan also acknowledged anger that his father had gotten out of the car. He had felt that his father's motivation was to fight the other driver and he was worried for his father's health and well‐being. He had not understood the legalities of exchanging insurance details after an accident, and though he had been interested in the altercation (he remembered turning music down in the car to listen in), he was unable to hear what was said, other than the other driver swearing. We updated his knowledge to include information about reporting of accidents, along with the sharing of insurance details for the purposes of sorting out the damages and responsibility. We used concepts he was familiar with as a means to help explain the process; so, for example, he was aware that school kept a record of injuries sustained at school and was able to work out that this information could be used to reduce commonly occurring accidents. In explaining the insurance, he was aware that damage to goods in a shop required the responsible party to pay for them; therefore, with cars costing considerably more, he was informed that insurance was a way to cover the eventuality of an accident and subsequent costs. Once he had integrated this knowledge, he was able to notice a reduction in his overall anxiety in the retelling as well as a reduction in his anger levels outside of sessions.

The final part of the narrative involved Dylan and his father arriving at his place of safety, which he had originally assumed was home. However, because of where the incident took place and the other driver's anger, Dylan's father had driven them to Dylan's grandparents’ house. In the updating of the narrative, Dylan's father acknowledged that this decision reflected his desire to report the incident, to calm himself, and for Dylan to have additional carer input to help him manage. While at Dylan's grandparents, Dylan's father called the police, who were aware of the incident because the ambulance that saw them at the scene had called it in. When the police arrived at the accident site, the two vehicles had already departed the scene. It was very helpful for Dylan to be able to acknowledge his father's need to calm down. Similarly, it was helpful for Dylan to acknowledge that his father had the same physiological response to a traumatic event as he himself had had, but that with more life experience, his father was able to process it and manage it slightly differently. Dylan had previously not been able to understand the police not visiting them, so we were able to educate him about incident reporting, and the fact that the insurance company would now deal with the situation so that no further contact with the other driver was required. In fact, the police had driven to the other driver's house and had seen the damage to his van; they also confirmed that the other driver was admitting liability and informed Dylan's father of this on the telephone while Dylan was in school.

Finally, we were able to update Dylan as to the progress of the insurance claim and the fact that the case was essentially closed from an insurance perspective. Dylan's father brought in a letter from the insurance company detailing the liabilities and explained that a payment had been received for the damage to the car, but compensation for personal injuries had not yet been finalized. Although Dylan was not able to read and understand all of this, it was tangible evidence of the process and it was agreed that when final payments were made, Dylan's father would let him know as a further update. All of this information was integrated into Dylan's narrative of the event as illustrated earlier.

### Outcome and Prognosis

**Figure 3:**
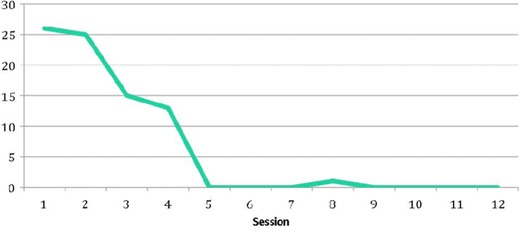
Child PTSD Symptom Scale (CPSS) scores through treatment. *Note*. Clinical cutoff is a CPSS score of 11.

Dylan worked very hard throughout his sessions and made good progress. Dylan's scores on a 0–10 Likert scale of distress during recounting the narrative had decreased as he had progressed with the process. He had indicated peak scores of 10 when discussing his physical pain, at the first recounting, and 8 when his father had got out of their car. However, by the end of the narrative sessions, he was not registering any discomfort or anxiety when reading through the narrative, talking about, or thinking about the trauma. He was reporting scores of 0 throughout these final sessions at all parts of the narrative. Dylan's scores also decreased on the Child PTSD Symptom Scale (CPSS; Foa, Johnson, Feeny, & Treadwell, 2001), from 26 at session 1 to 0 around session 5 and continuing at this level through to the his last session, following the trajectory shown in Figure 3.

At baseline, at the clinical interview, Dylan's father endorsed PTSD symptoms for Dylan of intrusions and reenactment in play, distress at reminders of the trauma, inability to recall details of the accident, negative beliefs, negative emotions, loss of interest, difficulty initiating sleep, irritability/anger, recklessness, concentration difficulties, and hypervigilance. At posttreatment, Dylan no longer met criteria for PTSD. The only endorsed PTSD symptom was distress at reminders of the trauma, and this was specifically reported as being concern over the presence of vans similar to the one involved in the accident.

### Summary

This case study demonstrates that the classic symptom cluster of PTSD can be seen in young children, that they can identify and work through their trauma narrative, and that they can update their memories and misappraisals using a cognitive methodology. More specifically, this case illustrates the application of the CBT‐3M approach to treating PTSD in very young children. It demonstrates qualitatively that young children are able to work cognitively, using developmentally appropriate techniques as outlined, to address inconsistencies and inaccuracies in their memories. They are able to confront distressing and painful memories while acknowledging stress reduction and less emotional demand as they do so.

It is hoped that the clinical trial (Dalgleish et al., 2015) of CBT‐3M in this age group will add significantly to the literature in terms of our understanding of how children develop PTSD after trauma, provide preliminary support for an efficacious treatment, and support our understanding of the cognitive model and its application to young children.

## References

[jclp22449-bib-0001] American Psychiatric Association . (1994). Diagnostic and statistical manual of mental disorders (4th ed.). Washington, DC: American Psychiatric Publishing.

[jclp22449-bib-0002] American Psychiatric Association . (2013). Diagnostic and statistical manual of mental disorders (5th ed.). Arlington, VA: American Psychiatric Publishing.

[jclp22449-bib-0003] Brewin, C. R. , Dalgleish, T. , & Joseph, S. (1996). A dual representation theory of posttraumatic stress disorder. Psychological Review, 103(4), 670–686. 8888651 10.1037/0033-295x.103.4.670

[jclp22449-bib-0004] Brewin, C. R. , & Holmes, E. A. (2003). Psychological theories of posttraumatic stress disorder, 23(3), 339–376. 10.1016/S0272-7358(03)00033-3 12729677

[jclp22449-bib-0005] Carpenter, G. L. , & Stacks, A. M. (2009). Developmental effects of exposure to Intimate Partner Violence in early childhood: A review of the literature. Children and Youth Services Review, 31(8), 831–839. 10.1016/j.childyouth.2009.03.005

[jclp22449-bib-0006] Cary, C. E. , & McMillen, J. C. (2012). The data behind the dissemination: A systematic review of trauma‐focused cognitive behavioral therapy for use with children and youth. Children and Youth Services Review, 34(4), 748–757. 10.1016/j.childyouth.2012.01.003

[jclp22449-bib-0007] Copeland, W. E. , Keeler, G. , Angold, A. , & Costello, E. J. (2007). Traumatic events and posttraumatic stress in childhood. Archives of General Psychiatry, 64(5), 577–584. 10.1001/archpsyc.64.5.577 17485609

[jclp22449-bib-0008] Dalgleish, T. (2004). Cognitive approaches to posttraumatic stress disorder: The evolution of multirepresentational theorizing. Psychological Bulletin, 130(2), 228–260. 14979771 10.1037/0033-2909.130.2.228

[jclp22449-bib-0009] Dalgleish, T. , Goodall, B. , Chadwick, I. , Werner‐Seidler, A. , McKinnon, A. , Morant, N. , … Meiser‐Stedman, R. (2015). Trauma‐focused cognitive behaviour therapy versus treatment as usual for post traumatic stress disorder (PTSD) in young children aged 3 to 8 years: Study protocol for a randomised controlled trial. Trials, 16(1), 116 10.1186/s13063-015-0632-2 25872653 PMC4417274

[jclp22449-bib-0010] Ehlers, A. , & Clark, D. M. (2000). A cognitive model of posttraumatic stress disorder. Behaviour Research and Therapy, 38(4), 319–345. 10761279 10.1016/s0005-7967(99)00123-0

[jclp22449-bib-0011] Foa, E. B. , Johnson, K. M. , Feeny, N. C. , & Treadwell, K. R. (2001). The Child PTSD Symptom Scale: A preliminary examination of its psychometric properties. Journal of Clinical Child Psychology, 30(3), 376–384. 10.1207/S15374424JCCP3003_9 11501254

[jclp22449-bib-0012] Lee, D. A. , Scragg, P. , & Turner, S. (2001). The role of shame and guilt in traumatic events: A clinical model of shame‐based and guilt‐based PTSD. British Journal of Medical Psychology, 74(4), 451–466. 11780793 10.1348/000711201161109

[jclp22449-bib-0013] Levendosky, A. A. , Huth‐Bocks, A. C. , Semel, M. A. , & Shapiro, D. L . (2002). Trauma symptoms in preschool‐age children exposed to domestic violence. Journal of Interpersonal Violence, 17(2), 150–164. 10.1177/0886260502017002003

[jclp22449-bib-0014] Lieberman, A. F. , & Knorr, K. (2007). The impact of trauma: A development framework for infancy and early childhood. Psychiatric Annals, 37(6), 416–422. 10.3928/0090-4481-20070401-10 17469301

[jclp22449-bib-0015] Meiser‐Stedman, R. (2002). Towards a cognitive‐behavioral model of PTSD in children and adolescents. Clinical Child and Family Psychology Review, 5(4), 217–232. 10.1023/A:1020982122107 12495267

[jclp22449-bib-0016] Meiser‐Stedman, R. , Dalgleish, T. , Glucksman, E. , Yule, W. , & Smith, P. (2009). Maladaptive cognitive appraisals mediate the evolution of posttraumatic stress reactions: A 6‐month follow‐up of child and adolescent assault and motor vehicle accident survivors. Journal of Abnormal Psychology, 118(4), 778–787. 10.1037/a0016945 19899847

[jclp22449-bib-0017] Meiser‐Stedman, R. , Dalgleish, T. , Smith, P. , Yule, W. , & Glucksman, E. (2007). Diagnostic, demographic, memory quality, and cognitive variables associated with acute stress disorder in children and adolescents. Journal of Abnormal Psychology, 116(1), 65–79. 10.1037/0021-843X.116.1.65 17324017

[jclp22449-bib-0018] Meiser‐Stedman, R. , Smith, P. , Glucksman, E. , Yule, W. , & Dalgleish, T. (2008). The posttraumatic stress disorder diagnosis in preschool‐ and elementary school‐age children exposed to motor vehicle accidents. American Journal of Psychiatry, 165(October), 1326–1337. 18676592 10.1176/appi.ajp.2008.07081282

[jclp22449-bib-0019] Meiser‐Stedman, R. , Smith, P. , McKinnon, A. , Dixon, C. , Trickey, D. , Ehlers, A. , … Dalgleish, T . (2016). Cognitive therapy as an early treatment for post‐traumatic stress disorder in children and adolescents: A randomized controlled trial addressing preliminary efficacy and mechanisms of action. Journal of Child Psychology and Psychiatry. 10.1111/jcpp.12673 PMC5362068 27976374

[jclp22449-bib-0020] Scheeringa, M. S. , & Haslett, N. (2010). The reliability and criterion validity of the Diagnostic Infant and Preschool Assessment: A new diagnostic instrument for young children. Child Psychiatry and Human Development, 41(3), 299–312. 10.1007/s10578-009-0169-2 20052532 PMC2862973

[jclp22449-bib-0021] Scheeringa, M. S. , Peebles, C. D. , Cook, C. A. , & Zeanah, C. H. (2001). Toward establishing procedural, criterion, and discriminant validity for PTSD in early childhood. Journal of the American Academy of Child and Adolescent Psychiatry, 40(1), 52–60. 10.1097/00004583-200101000-00016 11195563

[jclp22449-bib-0022] Scheeringa, M. S. , Weems, C. F. , Cohen, J. A. , Amaya‐Jackson, L. , & Guthrie, D. (2011). Trauma‐focused cognitive‐behavioral therapy for posttraumatic stress disorder in three‐through six year‐old children: a randomized clinical trial. Journal of Child Psychology and Psychiatry, and Allied Disciplines, 52(8), 853–560. 10.1111/j.1469-7610.2010.02354.x PMC3116969 21155776

[jclp22449-bib-0023] Scheeringa, M. S. , Zeanah, C. H. , & Cohen, J. A. (2011). PTSD in children and adolescents: toward an empirically based algorithma. Depression and Anxiety, 28(9), 770–782. 10.1002/da.20736 20734362 PMC6101653

[jclp22449-bib-0024] Scheeringa, M. S. , Zeanah, C. H. , Drell, M. J. , & Larrieu, J. A. (1995). Two approaches to the diagnosis of posttraumatic stress disorder in infancy and early childhood. Journal of American Academy of Child & Adolescent Psychiatry, 34(2), 191–200. 10.1097/00004583-199502000-00014 7896652

[jclp22449-bib-0025] Smith, P. , Perrin, S. , Yule, W. , & Clark, D. M. (2010). Post truamatic stress disorder: Cognitive therapy with children and young people. London; New York: Routledge.

[jclp22449-bib-0026] Smith, P. , Yule, W. , Perrin, S. , Tranah, T. , Dalgleish, T. , & Clark, D. M. (2007). Cognitive‐behavioral therapy for PTSD in children and adolescents: A preliminary randomized controlled trial. Journal of the American Academy of Child & Adolescent Psychiatry, 46(8), 1051–1061. 10.1097/CHI.0b013e318067e288 17667483

[jclp22449-bib-0027] Yule, W. , Bolton, D. , Udwin, O. , Boyle, S. , O'Ryan, D. , & Nurrish, J. (2000). The long‐term psychological effects of a disaster experienced in adolescence: I: The incidence and course of PTSD. Journal of Child Psychology and Psychiatry, and Allied Disciplines, 41(4), 503–511. 10836680

